# Efficacy of Killed Virus Vaccine, Live Attenuated Chimeric Virus Vaccine, and Passive Immunization for Prevention of *West Nile virus* Encephalitis in Hamster Model

**DOI:** 10.3201/eid0812.020229

**Published:** 2002-12

**Authors:** Robert B. Tesh, Juan Arroyo, Amelia P.A. Travassos da Rosa, Hilda Guzman, Shu-Yuan Xiao, Thomas P. Monath

**Affiliations:** * University of Texas Medical Branch, Galveston, Texas, USA; † Acambis, Inc., Cambridge, Massachusetts, USA

**Keywords:** West Nile virus, vaccines, infectious diseases, viral diseases, emerging diseases, disease prevention, arboviruses, ChimeriVax platform

## Abstract

Results of experiments evaluating the efficacy of three immunization strategies for the prevention of *West Nile virus* (WNV) encephalitis are reported. Immunization strategies evaluated included a killed virus veterinary vaccine, a live attenuated chimeric virus vaccine candidate, and passive immunization with WNV-immune serum; all were tested by using a hamster model of the disease. Each product protected the animals from clinical illness and death when challenged with a hamster-virulent wild-type WNV strain 1 month after initial immunization. The live attenuated chimeric virus vaccine candidate induced the highest humoral antibody responses, as measured by hemagglutination inhibition, complement fixation, and plaque reduction neutralization tests. Although the duration of protective immunity was not determined in this study, our preliminary results and the cumulative experience of other virus vaccines suggest that the live attenuated chimeric virus provides the longest lasting immunity.

After the appearance of *West Nile virus* (WNV) in North America and the resulting human and equine cases of encephalitis, considerable efforts have focused on developing vaccines against this emerging viral pathogen. A number of different WNV vaccine candidates have been recently described and are now in various stages of testing (1–4). A formalin-inactivated veterinary vaccine (West Nile Virus Vaccine, Killed, Fort Dodge Animal Health, Fort Dodge, IA) was conditionally licensed by the U.S. Department of Agriculture in August 2001 and has already been used in equines and exotic zoo birds in some areas of the country. We report the results of studies evaluating the efficacy of the killed veterinary vaccine, a live attenuated chimeric virus candidate, and passive immunization with immune serum for preventing WNV encephalitis in a hamster model of the disease (5,6).

## Materials and Methods

### Virus, Vaccines, and Immune Serum

 The virus used to infect animals in these studies was a second Vero cell passage of strain NY385-99, originally isolated from the liver of a Snowy Owl (*Nyctea scandiaca*) that died at the Bronx Zoo during the 1999 WNV epizootic in New York City (7). Two different WNV vaccines were evaluated in the hamster model: West Nile Encephalitis Virus Vaccine (killed), (Fort Dodge Animal Health, Fort Dodge, IA), a formalin-inactivated whole virus veterinary vaccine, and ChimeriVax–West Nile virus (Acambis, Inc., Cambridge, MA), a live attenuated chimeric virus vaccine candidate (1). The immune serum used in passive immunization experiments was prepared by pooling convalescent-phase serum samples of six hamsters that were bled 5 weeks after infection with WNV strain NY385-99.

### Hamsters and Hamster Model

 Animals used in these studies were adult (10–11 weeks old) female hamsters (*Mesocricetus auratus*) obtained from Harlan Sprague Dawley, Inc. (Indianapolis, IN). The hamster model of WNV encephalitis has been described (5,6). After intraperitoneal inoculation of 10^4^ 50% tissue culture infections dose_50_ (TCID_50_) of WNV strain NY385-99, fatal encephalitis developed in approximately 50% of adult hamsters. Animals were cared for in accordance with the guidelines of the Committee on Care and Use of Laboratory Animals (Institute of Laboratory Animal Resources, National Research Council) under an animal-care protocol approved by the University of Texas Medical Branch. All work with infected animals was carried out in biosafety level 3 facilities.

### Immunization Schedule and Challenge with WNV

 Hamsters immunized with West Nile Encephalitis Virus Vaccine (killed) received two intramuscular injections of 0.1 mL each, given 3 weeks apart, following manufacturer’s recommendation. Eleven days after the second immunization, the hamsters were bled to determine their antibody response; each animal was then inoculated intraperitoneally with 10^4^ TCID_50_ of WNV strain NY385-99. After challenge with live virus, the animals were bled daily for 6 consecutive days to measure the level of viremia and subsequent immune response. The animals were observed for another 21 days to determine if any developed signs of encephalitis or died.

 Two groups of hamsters were inoculated intramuscularly with the ChimeriVax–WNV vaccine. One group received 10^6.3^ PFU, and the other received 10^3.3^ PFU of the chimeric virus. Thirty-one and 32 days, respectively, after injection of the virus, the two groups of hamsters were inoculated intraperitoneally with 10^4^ TCID_50_ of WNV strain NY385-99. After challenge, the animals were bled daily for 6–7 days to measure the level of viremia and immune response, as described previously. These hamsters were also observed for 21 additional days for signs of illness or death.

 Two groups of hamsters also were passively immunized with different amounts (0.1 mL and 0.5 mL) of hamster WNV-immune serum, given intramuscularly. One day after passive immunization, both groups of animals were bled to determine if detectable titers of WNV hemagglutination inhibition (HI) antibodies were present in their serum. The animals then were inoculated intraperitoneally with 10^4^ TCID_50_ of WNV strain NY385-99, bled for 7 consecutive days, and observed for another 21 days, as described previously.

 A group of naïve (control) hamsters was also inoculated intraperitoneally with the same dosage of WNV strain NY385-99. Thirty-eight days after infection, 10 of the surviving animals were bled to determine their antibody response.

### Virus Titration and Antibody Determinations

 Serial blood samples from the hamsters were titrated in microplate cultures of the C6/36 clone of *Aedes albopictus* cells (8). The presence or absence of WNV viral antigen, determined by immunofluorescence, was used as the endpoint. This technique has been described in detail (5,6). WNV titers in the blood samples were calculated as the TCID_50_ per microliter of specimen by the method of Reed and Muench (9).

 Serum antibodies to WNV and *Yellow fever virus* (YFV) were measured by HI, complement fixation (CF), and plaque reduction neutralization (PRN) tests. Antigens for HI and CF tests were prepared from brains of newborn mice injected intracerebrally with the respective flaviviruses; these infected brains were treated by the sucrose-acetone extraction method (10). Hamster sera were tested by HI at serial twofold dilutions from 1:20 to 1:5,120 at pH 6.6 (WNV) or 6.4 (YFV) with 4 U of antigen and a 1:200 dilution of goose erythrocytes, following established protocols (10).

 CF tests were performed by a microtechnique (10) with two full units of guinea pig complement and antigen titers >1:32. Titers were recorded as the highest dilutions giving +3 or +4 fixation of complement on a scale of 0 to +4.

 PRN tests on hamster serum were performed by a previously described technique (11) in 24-well, Vero-microplate-cell cultures, using a fixed virus inoculum (~100 PFU) against varying serum dilutions (1:10 to 1:20,480). For PRN tests, the Egypt 101 strain of WNV (12) was used because this strain produced larger and sharper plaques than NY385-99. Hamster serum samples were diluted in phosphate-buffered saline, pH 7.4, containing 10% fresh guinea pig serum. Virus inoculum was mixed with an equal volume of each serum dilution; and the mixture was incubated overnight at 4°C. The following day, 50 uL of the serum-virus mixture was injected into Vero microplate cultures, with two wells per serum dilution. Virus plaques were read 4 days later; >90% plaque reduction was used as the endpoint.

## Results

### WNV Infection in Naïve Hamsters

 The level and duration of viremia, antibody response, and deaths in naïve (non-immune) adult hamsters after WNV infection have been described (5,6). Following intraperitoneal inoculation of 10^4^ TCID_50_ of WNV strain NY385-99, moderate levels of viremia that persisted for 6 days developed in the hamsters (Figure). HI antibodies were detected in the animals as early as day 5, and titers continued to increase through day 7. Initially, HI antibody response in primary WNV infection is specific; but after 3 or 4 weeks, the antibody pattern becomes more broadly reactive and serologic cross-reactions occur with other flavivirus antigens (6). Table 1 shows the HI, CF, and PRN antibody responses to WNV antigen and virus in 10 naïve adult hamsters that survived infection with the NY385-99 virus strain. These animals were bled 38 days after infection. Hamsters who survived infection with wild-type WNV appeared to have solid immunity 1 month after infection (Table 1). Convalescent-phase sera from some of these animals were used to prepare the WNV immune serum used in the passive immunization experiments described below.

**Figure:**
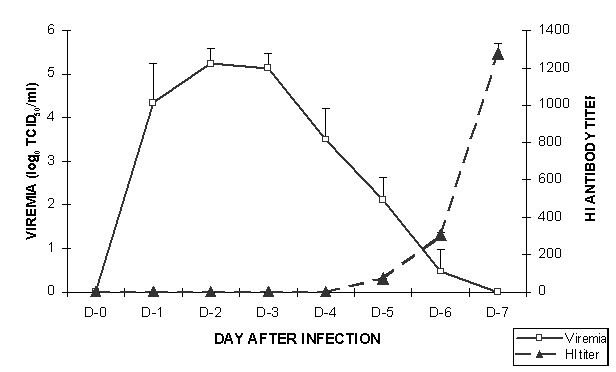
Daily mean (plus or minus the standard deviation) virus titers and hemagglutination inhibition (HI) antibody levels in 10 naïve (control) hamsters after intraperitoneal inoculation of 10^4^ TCID_50_ *West Nile virus* strain NY385-99.

**Table 1:** Hemagglutination inhibition, complement fixation, and plaque reduction neutralization antibody responses of naïve adult golden hamsters that survived *West Nile virus* infection^a,b^

Animal no.	HI antibody titer WNV antigen	CF antibody titer WNV antigen^c^	WNV PRN antibody titer^d^
H-8589	1:1280	1:320	1:10,240
H-8590	1:640	1:160	1:10,240
H-8591	1:640	1:320	1:5120
H-8592	1:640	1:80	1:2560
H-8593	1:640	1:160	1:2560
H-8594	1:1280	1:160	1:5120
H-8595	1:1280	1:320	1:10,240
H-8596	1:1280	1:320	>1:20,480
H-8597	1:1280	1:320	1:5120
H-8598	1:1280	1:640	1:2560

^a^HI, hemagglutination inhibition; CF, complement fixation; PRN, plaque reduction neutralization; WNV, *West Nile virus*. ^b^Animals 38 days after inoculation of virus strain NY385-99 (10^4.0^ 50% tissue culture infective dose_50_ given intraperitoneally). ^c^WNV antigen titer = >1:32. ^d^Highest serum dilution producing >90% plaque inhibition.

### WNV Infection in Hamsters Previously Immunized with a Killed Vaccine

Table 2 shows the HI, CF, and PRN antibody responses of nine hamsters 32 days after immunization (2 injections) with the Fort Dodge WNV killed vaccine. One month after the initial immunization, eight of nine animals had detectable levels of HI and CF antibodies; five of nine hamsters had low levels of WNV-neutralizing antibodies. Six days after challenge with the wild-type virus, most of the hamsters had an increase (range two-fold to 32-fold increase) in their HI and CF antibody titers, indicating some degree of antigenic stimulation and possible virus replication. Two of the hamsters had detectable levels of viremia after challenge with the wild-type virus (Table 3). However, none of the animals appeared clinically ill, and all survived.

**Table 2:** WNV antibody response of hamsters after immunization with WNV killed virus vaccine (Fort Dodge) and challenge with the NY385-99 strain of WNV^a,b^

Animal no.	HI antibody titer	CF antibody titer	PRN antibody titer^c^
32 days after Ft. Dodge vaccine			
H-8440	1:40	1:40	1:20
H-8441	0^d^	0	<1:10
H-8442	1:20	1:40	<1:10
H-8443	1:40	1:40	1:20
H-8445	1:40	1:40	1:20
H-8446	1:20	1:20	1:40
H-8447	1:20	1:20	<1:10
H-8448	1:20	1:20	<1:10
H-8449	1:40	1:20	1:10
6 days after challenge with WNV			
H-8440	1:80	1:80	ND
H-8441	1:320	1:160	ND
H-8442	1:40	1:40	ND
H-8443	1:40	1:40	ND
H-8445	1:320	1:160	ND
H-8446	1:80	1:40	ND
H-8447	1:320	1:160	ND
H-8448	1:160	1:80	ND
H-8449	1:160	1:40	ND

^a^WNV, *West Nile virus*; HI, hemagglutination inhibition; CF, complement fixation; PRN, plaque reduction neutralization; ND, not done. ^b^10^4.0^ 50% tissue culture infective dose_50_ given intraperitoneally. ^c^Highest serum dilution producing >90% plaque inhibition. ^d^0 = <1:20.

**Table 3:** Level and duration of viremia in hamsters previously immunized with WNV killed virus vaccine (Fort Dodge) and challenged with the NY385-99 strain of WNV^a^

Animal no.	Postinfection, by day					
	1	2	3	4	5	6
8440	0^b^	0	0	0	0	0
8441	0	1.2	3.5	1.3	1.2	0
8442	0	0	0	0	0	0
8443	0	0	0	0	0	0
9445	0	0	0	0	0	0
8446	0	0	0	0	0	0
8447	0	0	0	0	0	0
8448	0	0.7	3.0	4.3	2.3	0
8449	0	0	0	0	0	0

^a^WNV, *West Nile virus*. ^b^Virus titers in blood expressed as log_10_ 50% tissue culture infective dose_50_/mL of blood. 0 = <10^0.7^ TCID_50_.

### WNV Infection in Hamsters Previously Immunized with Live, Attenuated Chimeric Vaccine

Tables 4 and 5 show the antibody responses of hamsters receiving two different doses (10^6.3^ and 10^3.3^) of ChimeriVax-WNV vaccine. The results of these two experiments were similar. One month after immunization, all animals had detectable HI, CF, and PRN-antibody titers to WNV. When tested 6–7 days after challenge with the wild-type virus, none of the animals had a substantial change in antibody titer. WNV was detected in the blood of one animal on day 2; the titer was 10^0.7^ TCID_50_/mL (data not shown). None of the animals in these two groups appeared sick, and all survived.

**Table 4:** Antibody response of adult golden hamsters to intramuscular inoculation of 1.8 X 10^6^ of ChimeriVax–WNV and subsequent challenge with WNV (10^4.0^ TCID_50_ given intraperitoneally)^a^

Animal no.	HI antibody titer		CF antibody titer		WNV PRN antibody titer^b^
	YFV antigen	WNV antigen	YFV antigen	WNV antigen	
31 days after ChimeriVax-WNV					
H-8183	1:40	1:160	0^c^	0	1:160
H-8184	1:40	1:160	0	ND	1:160
H-8185	1:40	1:160	0	1:20	1:640
H-8186	1:80	1:320	0	1:40	1:320
H-8187	1:40	1:160	0	1:20	>1:640
H-8188	1:40	1:160	0	1:20	1:640
H-8189	1:80	1:320	0	1:40	1:640
H-8190	1:40	1:160	0	1:20	1:320
H-8191	1:80	1:160	0	1:20	1:80
H-8192	1:40	1:160	0	1:40	1:80
6 days after challenge with WNV					
H-8183	1:20	1:160	0	0	ND
H-8184	1:40	1:160	0	1:40	ND
H-8185	1:20	1:160	0	1:20	ND
H-8186	1:80	1:320	0	1:40	ND
H-8187	1:20	1:160	0	1:20	ND
H-8188	1:40	1:160	0	1:20	ND
H-8189	1:80	1:320	0	1:40	ND
H-8190	1:40	1:320	0	1:40	ND
H-8191	1:40	1:320	0	1:20	ND
H-8192	1:40	1:160	0	1:20	ND

^a^WNV, *West Nile virus*; HI, hemagglutination inhibition; CF, complement fixation; PRN, plaque reduction neutralization; YFV, *Yellow fever virus*; ND, not done. ^b^Highest serum dilution producing >90% plaque inhibition. ^c^0 = <1:20.

**Table 5:** Antibody response of adult golden hamsters to intramuscular inoculation of 1.8 X 10^3^ of ChimeriVax-WNV and WNV10^4.0^ TCID_50_ given intraperitoneally^a^

Animal no.	HI antibody titer			CF antibody titer		WNV PRN antibody titer^b^
	YFV antigen		WNV antigen	YFV antigen	WNV antigen	
32 days after ChimeriVax-WNV						
H-8322	1:40		1:160	0^c^	1:20	1:320
H-8323	1:20		1:80	0	1:40	1:160
H-8324	1:80		1:320	1:20	1:80	1:160
H-8325	1:40		1:320	0	1:80	≥ 1:640
H-8326	1:40		1:320	0	1:80	1:320
H-8327	1:40		1:320	ND	ND	1:320
H-8328	1:80		1:320	1:20	1:80	1:80
H-8329	1:40		1:320	0	1:80	1:80
7 days after challenge with WNV						
H-8322	1:40	1:320		0	1:20	ND
H-8323	1:40	1:160		0	1:20	ND
H-8324	1:80	1:320		0	1:40	ND
H-8325	1:80	1:320		0	1:80	ND
H-8326	1:80	1:320		0	1:40	ND
H-8327	1:40	1:160		0	1:20	ND
H-8328	1:80	1:320		0	1:40	ND
H-8329	1:40	1:320		0	1:80	ND

^a^WNV, *West Nile virus*; HI, hemagglutination inhibition; CF, complement fixation; PRN, plaque reduction neutralization; YFV, *Yellow fever virus*; ND, not done. ^b^Highest serum dilution producing >90% plaque inhibition. ^c^0 = <1:20.

### WNV Infection in Passively Immunized Hamsters

 Two groups of hamsters (A and B) were inoculated with 0.5 mL and 0.1 mL, respectively, of hamster WNV-immune serum (Table 6). HI antibody titer of the immune serum was 1:1,280; PRN titer was 1:5,120. Twenty-four hours later, the animals were bled and then injected with the wild-type virus. When tested 24 hours after receiving WNV-immune serum, all animals in group A had low but detectable HI antibody titers. No HI antibodies in group B were detectable after 24 hours. Seven days after challenge with WNV, one animal in group A still had detectable HI antibodies. Hamsters in groups A and B were bled for 6 consecutive days; no virus was detectable in any of the blood samples (data not shown). All animals in groups A and B appeared well and survived challenge. The absence of an antibody response or viremia in the passively immunized animals suggests that no virus replication occurred after challenge with WNV.

**Table 6:** WNV hemagglutination inhibition antibody titers in adult hamsters 24 h after inoculation (passive immunization) with WNV immune serum, and 7 days later after challenge with WNV (10^4.0^ TCID_50_ given intraperitoneally)^a^

Hamster no.	24 h after passive immunization	7 days after challenge with WNV
Group A – received 0.5 mL WNV immune serum		
H-8126	1:40^b^	0
H-8127	1:20	0
H-8128	1:20	0
H-8129	1:20	0
H-8130	1:20	0
H-8138	1:40	0
H-8139	1:40	1:10
Group B – received 0.1 mL WNV immune serum)		
H-8131	0	0
H-8132	0	0
H-8133	0	0
H-8134	0	0
H-8135	0	0
H-8136	0	0
H-8137	0	0

^a^WNV, *West Nile virus*. ^b^Hemagglutination inhibition antibody titer. 0 = <1:10.

## Discussion

 Each of the three immunization products evaluated in this study (killed whole virus vaccine, live attenuated chimeric virus vaccine, and passive immunization with immune serum) protected hamsters from clinical encephalitis and death upon subsequent challenge with the virulent wild-type WNV strain NY385-99. In contrast, fatal encephalitis developed in approximately 50% of naïve hamsters inoculated with the same virus dose (5). One obvious deficiency of our study was that the duration of protection induced by each immunization product was not determined. Determining the duration of protection is difficult with a relatively short-lived (approximately 2 yrs) animal such as a hamster; such studies are more meaningful when conducted by using longer-lived species such as horses or humans. Nonetheless, the general experience with other live and inactivated vaccines and the use of immune globulin for prevention of viral diseases offers some clue as to the advantages and disadvantages of the three approaches for preventing WNV encephalitis (13,14).

### Passive Immunization with WNV Immune Globulin

 Hamsters inoculated with WNV immune serum (0.5 mL and 0.1 mL) appeared to be completely protected when challenged with the wild-type virus 24 hours after passive immunization (Table 6). No virus was detected in the animals’ blood after challenge, and HI antibodies to WNV viral antigen did not develop. Immune globulins present in the WNV-immune serum probably inhibited virus replication; consequently, insufficient antigenic mass existed in the animals to stimulate an antibody response. One advantage of passive immunization with WNV-immune globulins is that the protective effect is almost immediate (<24 hours in the case of the hamsters in our experiment). Passive immunization with WNV-immune globulins might be desirable when rapid, temporary protection against the virus is needed or when a person with a compromised immune system requires protection. The major disadvantage of passive immunity acquired from immune globulins is the relatively short period of protection (13).

### Inactivated WNV Vaccine

 Most hamsters immunized with the Fort Dodge killed WNV vaccine had low levels of HI, CF, and PRN antibodies after two injections (Table 2). None of the animals that received the killed vaccine appeared clinically ill or died after challenge with the wild-type WNV. However, six of the nine hamsters had a substantial increase in their HI antibody titer after challenge with the wild-type virus; two of the nine animals subsequently had detectable viremia (Table 3). These data suggest that the immune response to the killed vaccine was insufficient to completely inhibit virus replication and that some degree of virus replication occurred after challenge with the wild-type virus.

 The Fort Dodge WNV veterinary vaccine used in this study is a commercially available formalin-inactivated whole virion preparation that has received conditional approval from the U.S. Department of Agriculture for use in horses. The first WNV vaccine approved for use in the United States, its substrate and degree of purification are not public information. Duration of protection with this vaccine is also unknown, although the manufacturer recommends that horses be immunized annually.

 The major advantage of killed vaccines is their safety; the disadvantages are that they often require multiple doses to elicit and sustain an effective immune response and that the immune response may be imbalanced, leading to subsequent potentiation of the disease (14,15). However, highly purified killed-virus vaccines have been used effectively in persons for the prevention of *Japanese encephalitis virus* and *Tick-borne encephalitis virus* (16,17).

### Live, Attenuated Chimeric WNV

 Hamsters receiving both doses (10^6.3^ or 10^3.3^ PFU) of the ChimeriVax-WNV had good HI and PRN-antibody responses to WNV, when tested 1 month after immunization (Tables 4 and 5). One animal (H-8183) did not develop CF antibodies after immunization, and two other hamsters were not tested. Notably, CF antibodies to WNV viral antigen developed in most of the hamsters after vaccination with the ChimeriVax-WNV, although humans without previous flavivirus exposure generally do not develop CF antibodies after administration of the 17D vaccine (18). The ChimeriVax technology platform uses YFV 17D as a live vector for envelope genes of WNV (1).

 Six and 7 days after challenge with the wild-type virus, we found no change in HI or CF titers of the animals previously immunized with the ChimeriVax-WNV (Tables 4 and 5). PRN titers were not tested after challenge. WNV was detected in the blood of a single animal on day 2, although WNV antibody titers did not increase in the blood of this hamster 6 days after challenge. These data suggest that minimal WNV replication occurred in the animals immunized with the chimeric virus when challenged with the wild-type virus. The levels of PRN antibodies present in ChimeriVax-WNV–immunized hamsters also suggest that the protection would be long lasting.

The major advantages of live attenuated virus vaccines are that they induce a more balanced immune response and that the resulting immunity is longer lasting than with killed vaccines or immune globulins (13–15). Major concerns with a live WNV vaccine are related to safety: 1) a potential vaccine might contain adventitious agents; 2) the vaccine virus might cause illness in some recipients or lose attenuation during manufacture or replication; and 3) stability. The second and third concerns are currently being investigated and addressed; results will be reported in subsequent publications. However, on balance, the ChimeriVax-WNV candidate vaccine appears to be quite effective in preventing WNV encephalitis, on the basis of our comparative studies in a hamster model of the disease.
